# Extracellular Vesicles from Adipose-Derived Mesenchymal Stem/Stromal Cells Accelerate Migration and Activate AKT Pathway in Human Keratinocytes and Fibroblasts Independently of miR-205 Activity

**DOI:** 10.1155/2017/9841035

**Published:** 2017-11-05

**Authors:** Andrea da Fonseca Ferreira, Pricila da Silva Cunha, Virgínia Mendes Carregal, Priscila de Cássia da Silva, Marcelo Coutinho de Miranda, Marianna Kunrath-Lima, Mariane Izabella Abreu de Melo, Camila Cristina Fraga Faraco, Joana Lobato Barbosa, Frédéric Frezard, Vivian Resende, Michele Angela Rodrigues, Alfredo Miranda de Goes, Dawidson Assis Gomes

**Affiliations:** ^1^Departamento de Bioquímica e Imunologia, Instituto de Ciências Biológicas, Universidade Federal de Minas Gerais, Belo Horizonte, MG, Brazil; ^2^Departamento de Fisiologia e Biofísica, Instituto de Ciências Biológicas, Universidade Federal de Minas Gerais, Belo Horizonte, MG, Brazil; ^3^Departamento de Cirurgia, Faculdade de Medicina, Universidade Federal de Minas Gerais, Belo Horizonte, MG, Brazil; ^4^Departamento de Patologia, Instituto de Ciências Biológicas, Universidade Federal de Minas Gerais, Belo Horizonte, MG, Brazil

## Abstract

Mesenchymal stem/stromal cells (MSCs) are promising tools in cell therapy. They secrete extracellular vesicles (EVs) that carry different classes of molecules that can promote skin repair, but the mechanisms are poorly understood. Skin wound healing is a complex process that requires the activity of several signaling pathways and cell types, including keratinocytes and fibroblasts. In this study, we explored whether adipose tissue MSC-derived EVs could accelerate migration and proliferation of keratinocytes and fibroblasts, activate the AKT pathway, and promote wound healing *in vivo*. Furthermore, we evaluated if EV effects are miR-205 dependent. We found that MSC EVs had an average diameter of 135 nm. Keratinocytes and fibroblasts exposed to EVs exhibited higher levels of proliferation, migration, and AKT activation. Topical administration of EVs accelerated skin wound closure. Knockdown of miR-205 decreased AKT phosphorylation in fibroblasts and keratinocytes, whereas migration was decreased only in keratinocytes. Moreover, knockdown of miR-205 failed to inhibit AKT phosphorylation in fibroblasts and keratinocytes exposed to EVs. About the mechanism of EV effects, we found that incubation with EVs prevented inhibition of AKT activation by miR-205 knockdown, suggesting that EVs activate AKT independently of miR-205. In conclusion, we demonstrated that EVs are a promising tool for wound healing.

## 1. Introduction

Mesenchymal stem/stromal cells (MSCs) can be isolated from many adult tissues [1]. Human adipose tissue was chosen as the source of stem cells in this study, due to its easy access and high mesenchymal stem cell population. The extraordinary plasticity of these cells was originally thought to contribute to their well-established therapeutic efficacy in a wide variety of disease models, as well as in human clinical trials. However, recent findings suggested that MSCs have a remarkable influence on the microenvironment by secreting a wide range of bioactive factors. This ability may contribute more to tissue repair than to their capacity for transdifferentiation [2].

MSC secretome comprises a wide variety of biomolecules, including cytokines, growth factors, and RNAs. These molecules could modulate the microenvironment and, thus, be responsible for the majority of beneficial effects of stem cells in tissue repair [3]. Recent studies indicated that MSCs also secrete extracellular vesicles (EVs) that contribute to cell-cell communication. After internalization, EVs may alter recipient cell fate by delivering transcriptional factors able to modulate cellular properties [4, 5].

On the other hand, wound repair is a complex process controlled by many secreted factors, including soluble proteins and RNAs [6]. Many preclinical and clinical studies have demonstrated that administration of MSCs accelerates wound closure, reduces scarring, and promotes collagen synthesis and angiogenesis [7, 8]. However, the mechanisms and factors responsible for the improvement of wound healing process during stem cell therapy are still poorly understood.

Several biochemical pathways coordinate skin integrity restoration [9]. The pathway regulated by miR-205 is involved in cell migration and proliferation. Lack of miR-205 results in epidermal defects caused by impaired proliferation, whereas genetic deletion of miR-205 leads to neonatal mortality in mice [10]. This microRNA modulates AKT activation and, therefore, increases migration in epithelial cells and promotes wound healing [11].

In this study, we evaluated the ability of adipose-derived MSC EVs to increase cell proliferation, migration, and AKT activation in human keratinocytes and fibroblasts and to promote wound healing *in vivo*. Furthermore, we examined whether EV effects on migration and activation of AKT were dependent on miR-205. Here, we demonstrated that EVs and its molecules are promising tools for wound healing.

## 2. Materials and Methods

### 2.1. MSC Isolation and Culture

MSCs were isolated from subcutaneous adipose tissue donated freely by lipoplasty surgery patients according to the regulations approved by the Universidade Federal de Minas Gerais's Research Ethics Committee (348/2016). Cell extraction was carried out as described by Zuk et al. [12]. Briefly, tissue samples were washed twice with phosphate-buffered saline (PBS, pH 7.4; Thermo Fisher Scientific, Waltham, MA, USA) to remove blood cells and incubated with 0.1% collagenase solution (Thermo Fisher Scientific) for 45 min at 37°C. After incubation, samples were centrifuged at 300 ×g for 7 min, the supernatant was discarded, and the remaining pellet was resuspended in Dulbecco's modified Eagle's medium (DMEM; cat. number: 12800-017, Thermo Fisher Scientific) containing 10% fetal bovine serum (FBS; Thermo Fisher Scientific) and 1% penicillin/streptomycin (PS; Sigma-Aldrich, St. Louis, MO, USA). Cells were transferred to culture flasks and kept in a humidified atmosphere of 95% air and 5% CO_2_ at 37°C. Culture medium was changed every 3 days.

### 2.2. Characterization of MSCs by Cytometry

MSCs (10^6^ cells per sample) were incubated with 0.4 *μ*g of primary antibodies against CD73 (Thermo Fisher Scientific, cat. number: 41-0200), CD90 (Thermo Fisher Scientific, cat. number: 14-0909-82), CD105 (Thermo Fisher Scientific, cat. number: 12-105742) (traditional membrane stem cell markers), CD19 (Thermo Fisher Scientific, cat. number: 11-0199-42), and CD45 (Thermo Fisher Scientific, cat. number: 11-9459-42) (hematopoietic cell markers) for 30 min at 4°C [13]. After washing, cells were incubated with a secondary antibody for 30 min at 4°C, washed again, and suspended in PBS. Alexa Fluor 488 goat anti-mouse (Thermo Fisher Scientific) was used for unconjugated primary antibodies. In control experiments, cells were incubated only with the secondary antibody to exclude nonspecific binding. Cytometry was performed using a Guava® EasyCyte™ 6–2 L (Millipore, Temecula, CA, USA). For each sample, at least 5000 events were acquired. Results were analyzed with FlowJo 7.6.5 software (LLC, Ashland, OR, USA).

### 2.3. Differentiation of Chondrocytes, Adipocytes, and Osteocytes

MSCs were incubated for 21 days in specific differentiation media, as previously described [12]. Following incubation, cells were fixed and stained by Von Kossa, oil red O, and alcian blue stains to visualize osteogenic, adipogenic, and chondrogenic differentiation, respectively. Although other strategies could be used to verify osteogenic and chondrogenic differentiation, Von Kossa and alcian blue stains are still regarded as good methods for that purpose. Next, cells were imaged using a QIClick camera (QImaging, Surrey, BC, Canada) coupled to an IX70 Olympus microscope controlled by Image-Pro Plus 7.0.1 (Media Cybernetics, Rockville, MD, USA).

### 2.4. EV Isolation

EVs were isolated as previously described [14]. Briefly, stem cells were incubated with serum-free DMEM for 48 h at 37°C, in the atmosphere of 95% air and 5% CO_2_. This step was necessary to assure that the medium collected had only EVs from stem cells, as serum-free DMEM does not contain EVs. After the incubation period, the medium underwent successive centrifugation steps. The first centrifugation was at 300 ×g for 7 min; then, the supernatant was centrifuged for 15 min at 1000 ×g, followed by another supernatant centrifugation for 40 min at 10000 ×g. Then, the supernatant was ultracentrifuged (100000 ×g for 70 min). Finally, the supernatant underwent the last ultracentrifugation (100000 ×g for 70 min), and the resulting pellet was composed of EVs. All centrifugation steps were carried out at 4°C. Cells used for EV extraction were in the fourth or fifth passage. For every complete experiment, EVs from stem cells from three different patients were isolated and used, totalizing 45 samples from 48 different patients in this study.

### 2.5. EV Characterization and Concentration

EVs isolated from serum-free DMEM as described above were resuspended in PBS and analyzed using NanoSight LM 10 system and Nanoparticle Tracking Analysis software (Malvern Instruments Ltd., Malvern, UK). We used 1.9 × 10^8^ vesicles in each experiment.

### 2.6. Isolation and Culture of Human Keratinocytes and Fibroblasts

Human keratinocytes were extracted as described by Zonari et al. [8]. Human skin samples were donated with consent from the patients that underwent abdominoplasty surgery according to regulations specified by the Universidade Federal de Minas Gerais Research Ethics Committee (55698116.2.0000.5149). The skin was separated from adipose subcutaneous tissue with scissors, and the fatty tissue was discarded. Then, skin samples were fragmented into 5 mm^2^ pieces and incubated in dispase solution (2.5 UI) for 16 h at 4°C. After this, the dermis and epidermis were separated with tweezers (fibroblasts were extracted from the dermis and keratinocytes—from the epidermis). The dermis was incubated in a 0.1% collagenase solution (Thermo Fisher Scientific) for 3 h at 37°C, and the epidermal fragments were incubated with 0.05% trypsin solution for 5 min at 37°C. After the incubation period, a cell scraper was used to remove the fragments of the epidermis to release keratinocytes. The resulting material was then passed through a 100 *μ*m filter to only collect the cells that were then placed in culture flasks with keratinocyte serum-free medium (KSFM) and cultured at 37°C in the atmosphere of 95% air and 5% CO_2_. After a 3 h incubation in collagenase solution, dermal fragments that remained undigested were discarded, and the supernatant underwent centrifugation for 7 min at 300 ×g. The resulting pellet was resuspended in DMEM containing 10% FBS and 1% PS and transferred to a culture flask. Cells were kept in a humidified atmosphere of 95% air and 5% CO_2_ at 37°C, and the medium was changed every three days.

### 2.7. Growth Curves

The growth curves were performed in keratinocytes and fibroblasts seeded in 24-well plates (5 × 10^4^ cells per well) and exposed to the medium with or without MSC EVs. The concentration of vesicles used was 3.16 × 10^7^ per mL and each well was exposed to one mL of enriched medium. Cells were counted in a Neubauer chamber daily for eight consecutive days. Cell counts were used to plot a growth curve.

### 2.8. Western Blot

For experiments with EVs, cells were exposed to enriched medium and the concentration of vesicles used were 3.16 × 10^7^ per mL. Initially, total protein samples were isolated from cells exposed to EVs using a sterile cell scraper and a lysis buffer (150 mM NaCl, 1 mM EDTA, 20 mM Tris–HCl, pH 8.0, 0.5% Nonidet P-40). Total protein concentration was quantified by the Bradford method (Bradford, 1973), and then, samples underwent electrophoresis. Proteins were then transferred to polyvinylidene fluoride membranes using a Trans-Blot® SD semidry transfer cell (BioRad, Hercules, CA, USA). Immunoblotting was performed by standard methods, as previously described [15]. Primary antibodies against total AKT (Cell Signaling Technology, Danvers, MA, USA; cat. number 9272, 1 : 1000), phospho-AKT (Cell Signaling Technology; cat. number 4060, 1 : 500), phospho-histone 3 (Sigma-Aldrich; cat. number MFCD01633984, 1 : 500), total histone H3 (Sigma-Aldrich; cat number MFCD01322157, 1 : 2000), Alix (Cell Signaling Technologies, cat. number: 2171), and CD9 (cat. number: MA1 80-307) were used. Band density analysis was performed using ImageJ software (https://imagej.nih.gov/ij/).

### 2.9. Scratch Wound Healing Assay

Human keratinocytes and dermal fibroblasts were seeded in 6-well plates at 80% confluence. After exposure to KSFM or DMEM (depending on the cell type) for 48 h, a scratch was made on the cell monolayer with a sterile 200 *μ*L pipette tip. Wells were washed with PBS and then exposed to MSC EVs. The concentration of vesicles used were 3.16 × 10^7^ per mL in each well. Scratch closure was monitored by imaging at 0, 12, and 24 h. Scratch areas were measured by Image-Pro Plus Software.

### 2.10. Excisional Wound-Splinting Model

All animal experiments were performed after obtaining an approval from the University Research Ethics Committee (168/2013). The excisional wound-splinting model was used as described by Wang and colleagues [16]. This model facilitates the approximation between rat and human skin wound healing by reducing skin traction. Briefly, 24 male Wistar rats (220 g) were divided into two groups: test group and negative control group. Animal groups were further divided into three subgroups of four animals, for assessments at 7, 14, and 21 days after exposure to gel preparations. Rats were anesthetized using an intraperitoneal injection of ketamine and xylazine. Excision wounds (5 mm in diameter) were made on rat dorsal skin with a mini punch. Sterile silicone rings were glued around the wounds with a high-performance glue. Animals were placed in individual cages and received aspirin for pain relief. Throughout the experiment, they had free access to food and water.

### 2.11. Gel with EVs

Hydroxyethyl cellulose aqueous gel (2%) was made in sterile conditions and admixed to EV suspension (1.9 × 10^8^ vesicles) in a 1 : 1 ratio. The resulting 1% hydroxyethyl cellulose gel comprising MSC-derived EVs was aseptically applied daily on wounds of animals in the test group, whereas animals from the control group were treated with plain 1% hydroxyethyl cellulose gel without EVs. Wound parameters were assessed at 7, 14, and 21 days after the wound was made. Images were taken every day.

### 2.12. Real-Time PCR

Human keratinocytes and dermal fibroblasts were seeded in 6-well plates (5 × 10^5^ cells per well). RNA was extracted with a mirVana® RNA extraction kit (Thermo Fisher Scientific) and used for reverse transcriptase reaction. We used a TaqMan probe kit (Thermo Fisher Scientific, cat. number 4427975); amounts of RNA and cDNA were as recommended by the manufacturer. The reactions were read using a 7900HT Fast Real-Time PCR System and analyzed with SDS 2.4 software (Thermo Fisher Scientific). Relative gene expression was calculated by the 2^−(ΔΔCt)^ method [17].

### 2.13. Transfection of siRNA

Transfection was conducted as recommended by the manufacturer. Human dermal fibroblasts and keratinocytes were seeded at 60–70% confluence and then exposed to a suspension of Lipofectamine 2000 (Thermo Fisher Scientific) with 25 nM siRNA in Opti-MEM (Thermo Fisher Scientific) for 5 h. The siRNAs used were all purchased from Thermo Fisher Scientific: mirVana miRNA inhibitor, hsa-miR-205-5p (assay ID: MH11015; catalog number: 4464084); mirVana miRNA mimic, hsa-miR-205-5p (assay ID: MC11015, catalog number: 4464066); and mirVana miRNA inhibitor negative control number 1 (catalog number: 4464076). After the incubation, the transfection medium was discarded and replaced by DMEM or KSFM, depending on the cell type. Tests were performed after 48 h.

### 2.14. Statistical Analysis

The data shown represent at least three independent experiments and are expressed as the mean ± standard deviation. The statistical analyses were performed with GraphPad software. The experiments with more than two data groups were compared by using one-way ANOVA and the Bonferroni posttest. *P* values of at least <0.05 were considered to be statistically significant.

## 3. Results and Discussion

### 3.1. Characterization of MSCs

**Figure 1:**
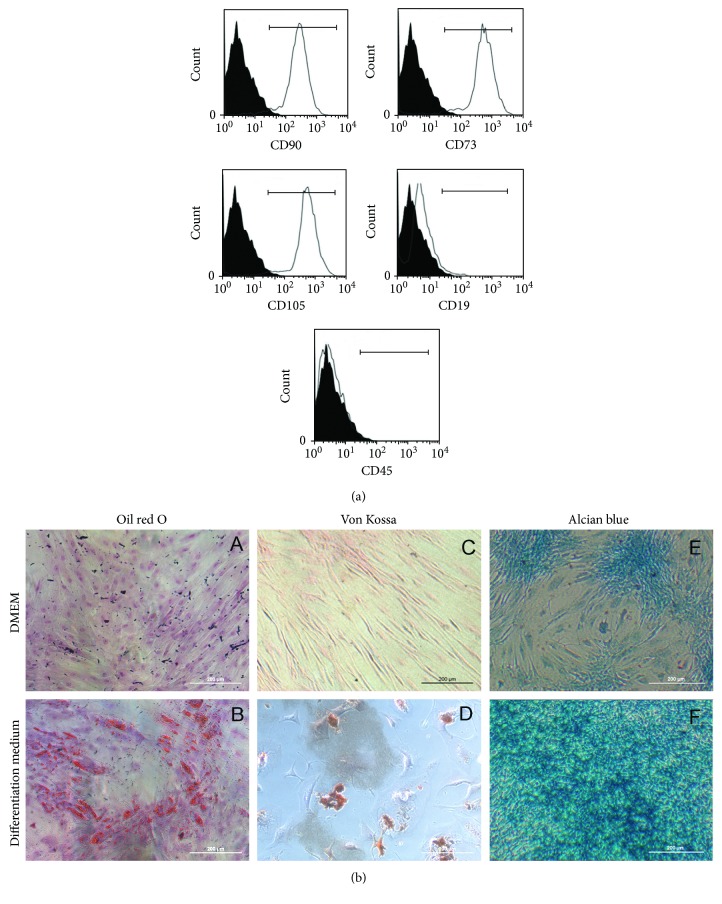
MSC characterization. (a) Cytometry graphs in solid black curves refer to the negative controls (cells incubated with secondary antibody) and nonfilled curves indicate the marker tested. The cell population expressed CD90, 73, and 105 and did not express CD19 and 45. (b) Brightfield images are showing that MSCs are able to differentiate in adipocytes, osteocytes, and chondrocytes. Undifferentiated cells were grown only in DMEM (A, C, and E) and show only background staining for oil red O on (A), Von Kossa on (C), and alcian blue on (E). Once incubated in differentiation-inducing culture mediums, adipose-derived MSCs showed positive staining for oil red O on (B), Von Kossa on (D), and for alcian blue on (F). Scale Bar = 200 *μ*m. Representative images of three biological replicates that were conducted independently.

Initially, MSCs were characterized according to the three criteria proposed by the Mesenchymal and Tissue Stem Cell Committee of the International Society for Cellular Therapy [18]. First, cell-surface antigen profile was ascertained by staining the cells with specific monoclonal antibodies, followed by cytometry analyses (Figure 1). We investigated the expression levels of cell markers CD90 (91.4% ± 0.1%), CD73 (92.9% ± 0.1%), CD105 (92% ± 0.1%), CD19 (4.9% ± 0.1%), and CD45 (1.3% ± 0.1%). These percentages of labeled cells indicated acceptable culture purity according to the criteria proposed by Bourin et al. [18]. MSCs adhered to the plastic material of the plates and showed fibroblast-like morphology (polygon-like or spindle-like with processes, Figure 1(b)). Finally, MSCs were capable of differentiating into adipocytes, osteoblasts, and chondrocytes (Figure 1(b)). After 21 days of exposure to differentiation medium followed by oil red O, Von Kossa, or alcian blue staining, it was possible to observe differentiation of stem cells into different types of cells, which displayed fat droplets in their cytoplasm, mineral deposits, and proteoglycan inclusions, respectively. These results suggested that the cell population isolated from subcutaneous adipose tissue in this study indeed comprised true MSCs, classified according to the criteria of the Tissue Stem Cell Committee of the International Society for Cellular Therapy [18].

### 3.2. Characterization of EVs Isolated from MSCs

**Figure 2:**
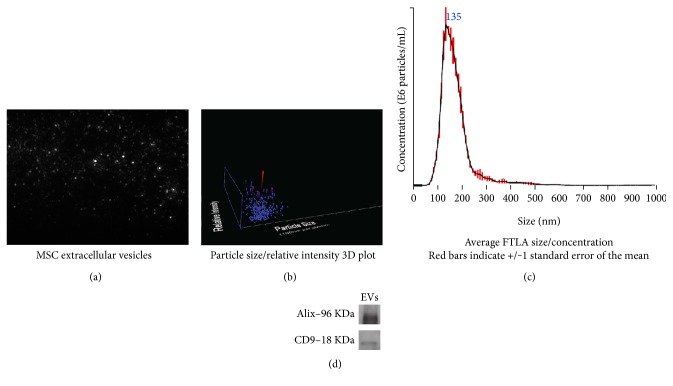
Physical characteristics of extracellular vesicles derived from mesenchymal stem/stromal cells. (a) Snapshot of extracellular vesicles derived from mesenchymal stem/stromal cells in solution shown as gray dots on a black background. (b) 3D plot of extracellular vesicle size distribution. The red dot represents vesicles with the predominant diameter. (c) 2D plot of extracellular vesicle size distribution. Average diameter size is 135 nm. (d) Western blot for two proteins present in EVs, Alix, and CD9. Representative images of at least three biological replicates that were conducted independently.

Next, EVs were analyzed by a NanoSight LM 10 system, using Nanoparticle Tracking Analysis (NTA) software. Results of these experiments are shown in Table 1 and in Figure 2. The average diameter of the vesicles observed in our preparations was 135 nm. We also determined their density, spreading speed, and sample viscosity. As shown in Figure 2, the curve base is broad, indicating that there is variation in size within the population. Accumulating data support the notion that the content, size, and membrane composition of EVs are highly heterogeneous and dynamic, depending on the cellular source, state, and environmental conditions [19, 20]. Also, we performed Western blot for Alix and CD9, two proteins present in EVs. Many efforts have been made by the scientific community to isolate EV subtypes, but the results were inconclusive. Hence, in this work, we assumed that we dealt with a mixture of different EV populations [19].

### 3.3. EVs Increased Proliferation of Fibroblasts and Keratinocytes

**Figure 3:**
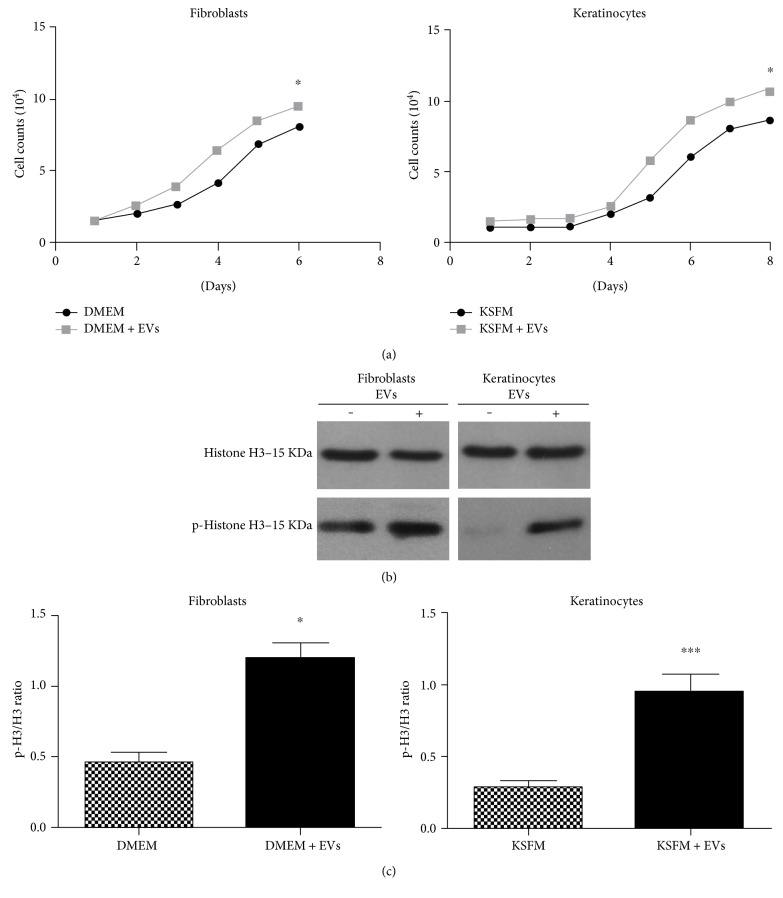
Extracellular vesicles derived from mesenchymal stem/stromal cells induce proliferation of fibroblasts and keratinocytes. (a) Growth curves comparing proliferation of fibroblasts (left graph) and keratinocytes (right graph) cultured in medium (DMEM or KSFM depending on cell type) in the absence (medium) or presence of extracellular vesicles (medium + EVs) derived from mesenchymal stem/stromal cells (MSCs). (b) A representative Western blot gel illustrating bands for total and phosphorylated histone H3 (p-histone H3). (c) Densitometry analysis of the p-H3/total H3 ratio. Incubation with MSC EVs was associated with higher relative levels of phosphorylated (activated) histone H3 in both fibroblasts and keratinocytes. Data are presented as the mean ± standard deviation of three biological independent experiments. Statistical significance of differences is indicated as follows: ^∗^*P* < 0.05; ^∗∗∗^*P* < 0.001.

To assess whether MSC EVs could induce proliferation of fibroblasts and keratinocytes *in vitro*, cells of both types were exposed to MSC EVs and two kinds of experiments were conducted. As shown in Figure 3(a), growth curves of fibroblasts and keratinocytes indicated that MSC EVs increased cell proliferation, which would be a beneficial factor for wound healing [9, 21]. Western blot analysis showed greater phosphorylation of histone H3, a mitosis marker [22], in cells of both types when exposed to MSC EVs (Figures 3(b) and 3(c)). Those results indicated that EVs modified epithelial cell proliferation. Previously, it has been reported that EVs from stem cells induced cell proliferation by modulating microenvironments and promoting mitosis [23, 24]. As cell proliferation is a key step in skin wound healing, and its impairment is directly associated with the formation of chronic wounds, the capacity of stem cell EVs to accelerate this process may be a useful therapeutic feature.

### 3.4. EVs Induced Migration of Fibroblasts and Keratinocytes

**Figure 4:**
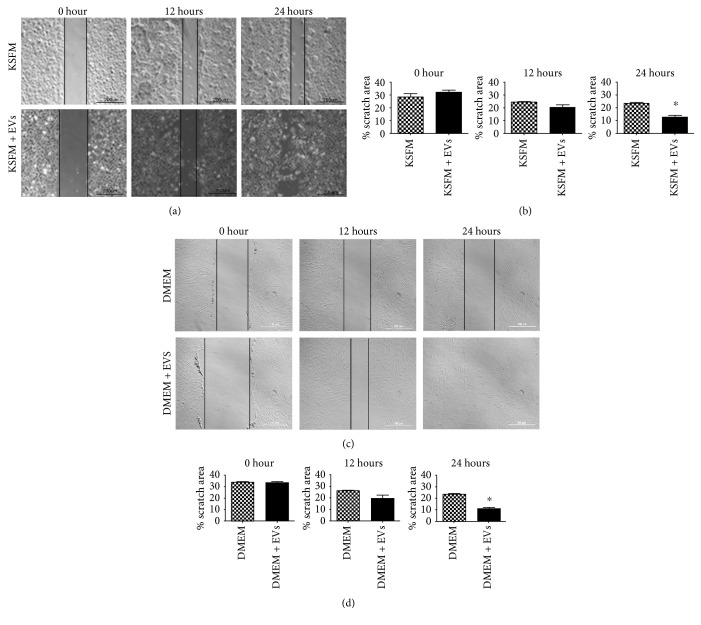
Extracellular vesicles derived from mesenchymal stem/stromal cells induce keratinocyte and fibroblast migration. (a) Phase contrast images of human keratinocytes cultured in DMEM in the absence (KSFM) or presence of extracellular vesicles (KSFM + EVs) derived from mesenchymal stem/stromal cells (MSCs) in 0, 12, and 24 h after scratch. Scale bar = 200 *μ*m. (b) Relative areas affected by the scratch wound at the three time points of the experiments. Significantly smaller scratch wound area was observed in 24 h after the scratch following the exposure to MSC EVs, indicating faster keratinocyte migration. Data are presented as the mean ± standard deviation of three independent experiments. Statistical significance of differences is indicated as follows: ^∗^*P* < 0.05. (c) Phase contrast images of human fibroblasts cultured in DMEM in the absence (DMEM) or presence of extracellular vesicles (DMEM + EVs) derived from mesenchymal stem/stromal cells (MSCs) in 0, 12, and 24 h after scratch. Scale bar = 200 *μ*m. (d) Relative areas affected by the scratch wound at the three time points of the experiments. Significantly smaller scratch wound area was observed in 24 h after the scratch following the exposure to MSC EVs, indicating faster fibroblast migration. Data are presented as the mean ± standard deviation of three biological independent experiments.

Epidermal keratinocytes and dermal fibroblasts were used in scratch wound healing assays, as described in Materials and Methods. As shown in Figure 4, the presence of EVs in the culture medium was associated with a more rapid decrease in the area affected by the initial wound, indicating increased migration after 24 h of exposure to MSC EVs in cells of both types. Cell migration in the wound bed is essential for skin wound healing, and it is known to be affected in some chronic conditions, for example, in diabetic chronic wounds [25]. It has been suggested that one of the reasons of skin cell migration impairment in chronic wounds is the depletion of stem cells in those areas, which creates imbalance in the microenvironment and decelerates wound healing process. It will be interesting to substantiate in more direct experiments whether MSC EVs and their signals can indeed modulate cell migration, as suggested by our results (Figure 4) and by data from other groups [23, 24].

### 3.5. EVs Accelerated Wound Healing

**Figure 5:**
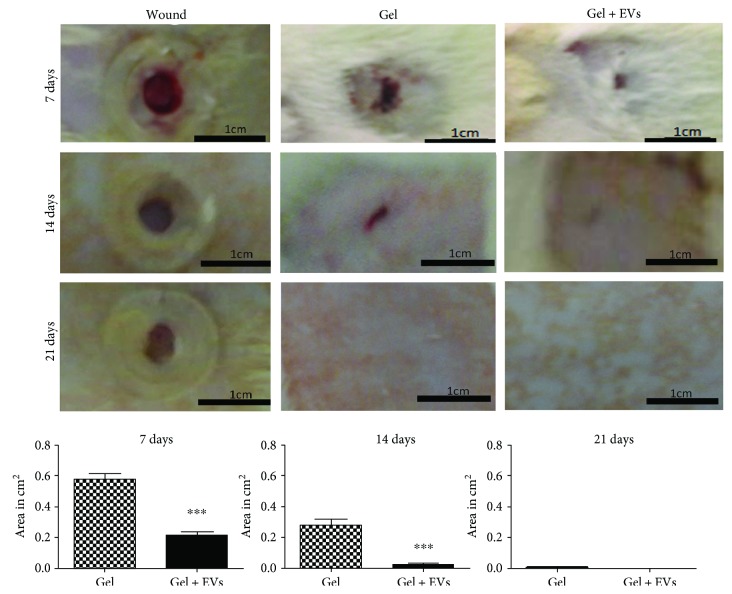
Gel enriched with extracellular vesicles derived from mesenchymal stem/stromal cells accelerated wound healing in rat excisional wound-splinting model. (a) Representative photographs of rat skin wounds that were either untreated (left column), exposed to hydroxyethyl cellulose gel (middle column), or exposed to hydroxyethyl cellulose gel enriched with extracellular vesicles (EVs) derived from mesenchymal stem/stromal cells at different time points of the experiment (7, 14, and 21 days after the wound was created). (b) Comparison of relative wound areas between negative control (gel) and test groups (gel + EVs). Data are presented as the mean ± standard deviation of three biological independent experiments. Statistical significance of differences is indicated as follows: ^∗∗∗^*P* < 0.001.

Male Wistar rats were subjected to the excisional wound-splinting procedure and then exposed to aqueous hydroxyethyl cellulose gel with or without MSC EVs for 7, 14, and 21 days. We found that treated animal wounds closed more rapidly than control wounds (Figure 5(a)). Furthermore, we observed that in rats treated with the gel enriched with MSC EVs, wound closure occurred at a faster rate than in animals treated with EV-free gel (Figure 5(b)). Acceleration of wound healing by stem cell-derived EVs has been reported previously. However, in those experiments, more invasive treatments, namely, intradermal injection of stem cell EV preparations were used [26]. In this work, we demonstrated comparable acceleration of wound healing by topical application of MSC EVs, which is a more advantageous setting that causes less therapeutic discomfort. Our results seem to be in accordance with suggestions of other authors about a promising potential of MSC EVs in skin wound healing [26, 27].

### 3.6. EVs Promoted AKT Phosphorylation in Fibroblasts and Keratinocytes

**Figure 6:**
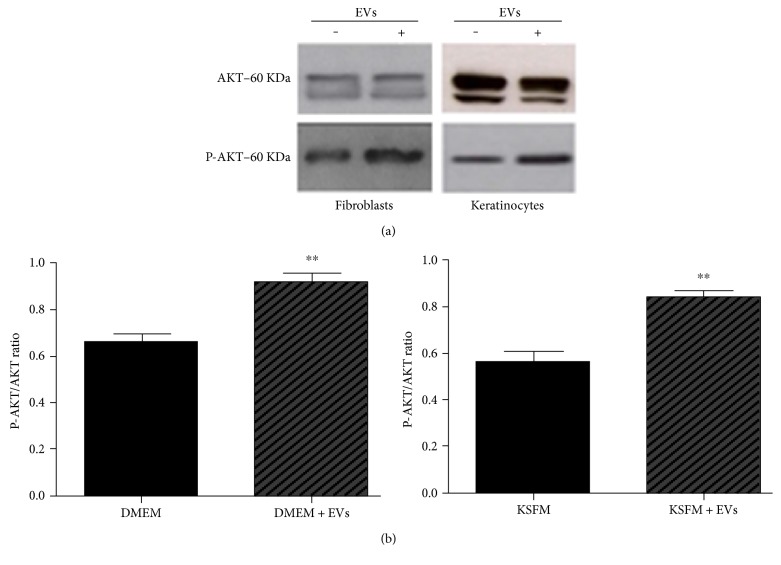
Exposure to extracellular vesicles derived from mesenchymal stem/stromal cells induced AKT phosphorylation in human keratinocytes and fibroblasts. (a) A representative Western blot gel illustrating bands for total and phosphorylated AKT (p-AKT) in cells before and after exposure to extracellular vesicles (EVs) derived from mesenchymal stem/stromal cells (MSCs). (b) Densitometry analysis of the p-AKT/total AKT ratio. Incubation with MSC EVs was associated with higher relative levels of phosphorylated (activated) AKT in both fibroblasts (left graph) and keratinocytes (right graph). Data are presented as the mean ± standard deviation of three biological independent experiments. Statistical significance of differences is indicated as follows: ^∗∗^*P* < 0.01.

The AKT pathway is considered one of the major biochemical pathways regulating migration of epithelial cells. To assess whether MSC EVs induced AKT activation, Western blot assays were performed in fibroblasts and keratinocytes. As shown in Figure 6, exposure of keratinocytes and fibroblasts to MSC EVs was associated with higher levels of phosphorylated AKT, which would increase the overall activity of the AKT pathway. Sometimes, there are some double bands in the Western blots for total AKT. The heaviest band was used for quantifications that was the band always present in the phosphorylated form. Recently, other authors found that AKT phosphorylation was higher in skin cells exposed to MSC EVs [10, 11]. Those results are interesting from the therapeutic point of view, as modulation of the AKT pathway activity may be a promising avenue of research into novel stimulators of wound healing process.

### 3.7. Exposure of Fibroblasts and Keratinocytes to EVs Increased miR-205 Expression

**Figure 7:**
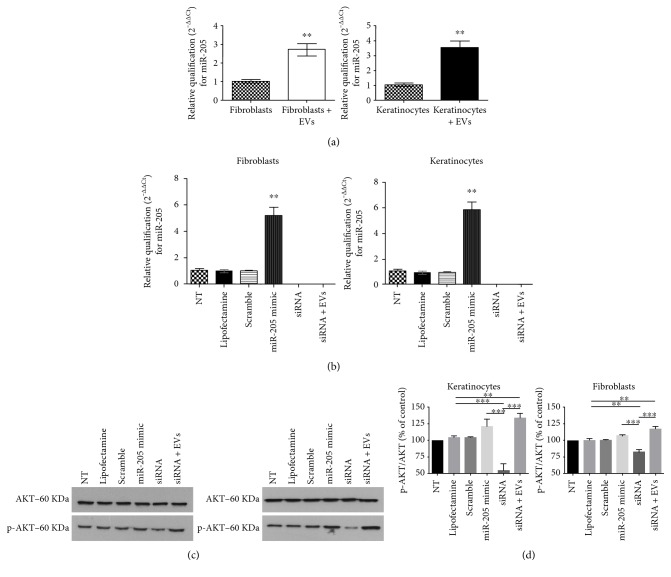
Effects of extracellular vesicles derived from mesenchymal stem/stromal cells on AKT phosphorylation are independent of miR-205. (a) Graphs illustrate relative levels of miR-205 in human fibroblasts and keratinocytes cultured in standard culture medium or in the medium containing EVs derived from mesenchymal stem/stromal cells (MSCs). Relative expression of miR-205 was higher in cells exposed to MSC EVs. qPCR data were normalized using the 2^−ΔΔCt^ method. Data are presented as the mean ± standard deviation of three independent experiments. Statistical significance of differences is indicated as follows: ^∗∗^*P* < 0.01. (b) Quantification of miR-205 relative expression levels by qPCR in fibroblasts (left graph) and keratinocytes (right graph) from the following experimental groups: nontreated (NT), exposed only to lipofectamine only (lipofectamine), transfected with scramble miR (scramble), transfected with synthetic miR-205 (miR-205 mimic), transfected with siRNA for miR-205 (siRNA), and, finally, transiently silenced for miR-205 and exposed to extracellular vesicles from mesenchymal stem/stromal cells (siRNA + EVs). Data are presented as the mean ± standard deviation. Statistical significance of differences is indicated as follows: ^∗∗^*P* < 0.01. (c) Representative Western blot gels illustrating bands for total and phosphorylated AKT (p-AKT) from experimental groups described previously. (d) Densitometry analysis of the p-AKT/total AKT ratio in lysates of cells from different experimental groups. Data are presented as the mean ± standard deviation of three biological independent experiments. Statistical significance of differences is indicated as follows: ^∗∗^*P* < 0.01; ^∗∗∗^*P* < 0.001.

It has been shown that one of the most abundant microRNAs in the skin, miR-205, is an important modulator of AKT pathway activity [11]. Moreover, we found the presence of miR-205 in EV samples from MSCs, through the Next Generation Sequencing experiments (data not shown). To evaluate whether MSC EVs modulated miR-205 expression, qPCR experiments were conducted in fibroblasts and keratinocytes after exposure to EVs for 24 h. We found that treatment with MSC EVs led to a ~3-fold increase in miR-205 expression level (Figure 7(a)). Although miR-205 is present inside the EVs from stem cells, we could not assume at this point that the increased expression observed in qPCR experiments is strictly due to the transfer from EVs to cells with the experiments described here. As widely known, microRNA biology is complex and could be modulated by several factors, including other microRNAs [10, 11].

Next, we carried out qPCR experiments to verify expected changes in cellular miR-205 levels after its overexpression or knockdown. Transfection of 25 nM of miR-205 sequence increased the expression level of this microRNA ~ 6-fold in both fibroblasts and keratinocytes (miR-205 mimic). We also examined the consequences of miR-205 knockdown in cells before (siRNA group) and after exposure to EVs (siRNA + EVs). We achieved 100% knockdown in both cell groups transfected with siRNA. Expression levels of miR-205 in cells exposed only to the transfection reagent lipofectamine (lipofectamine group) or lipofectamine plus a scramble sequence (scramble group) did not change, as expected (Figure 7(b)).

We also examined the effects of miR-205 overexpression and knockdown on AKT activation by performing Western blot analysis of the levels of phosphorylated and total AKT (Figure 7(c)). In human fibroblasts and keratinocytes, miR-205 overexpression did not induce significant AKT phosphorylation compared to the levels detected in cells treated with lipofectamine or scramble RNA. This result contrasted with the data reported by Yu et al. [11] who showed that miR-205 could induce AKT activation in keratinocytes. This could be attributed to the miR-205 concentration used. We decided not to increase the concentration of siRNA because we already had a 6-fold change in cells transfected with miR-205 mimic sequence that was around twice the amount of miR-205 found in the cells exposed to EVs. However, miR-205 knockdown led to lower levels of phosphorylated AKT in cells of both types, as previously demonstrated.

Properties of fibroblasts and keratinocytes exposed to MSC EVs were similar when miR-205 was silenced. They exhibited higher levels of AKT phosphorylation than cells treated with lipofectamine only. These results indicated that AKT activation upon exposure to MSC EVs was likely independent of miR-205 expression. Considering the complexity of EV cargo, it is known that other molecules present in the vesicles could work collectively, modulating the microenvironment, favoring migration in skin cells. We are currently analyzing others RNAs present to the EVs to further investigate other potential targets for wound healing process. Also, several growth factors could be present in the EVs. Perhaps many molecules could participate in the effects described in this paper.

**Figure 8:**
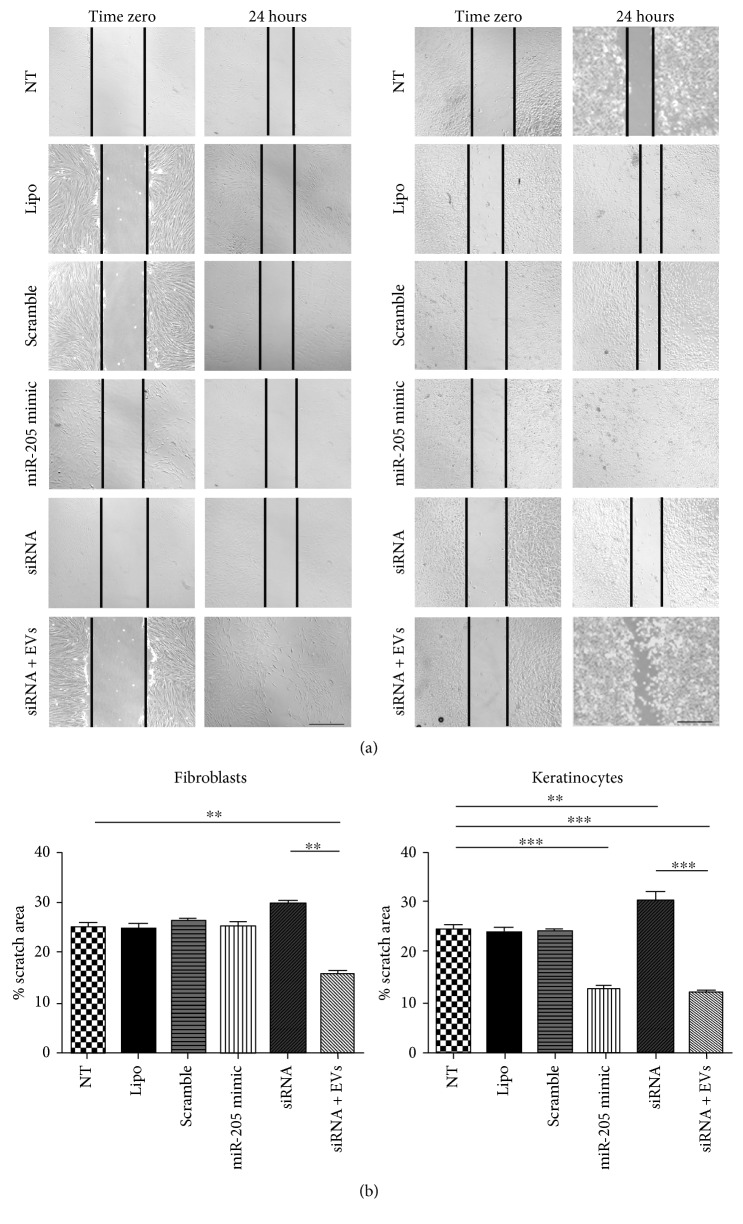
Effects of extracellular vesicles on the migration of fibroblasts and keratinocytes do not require miR-205. (a) Phase contrast images of scratch wound straight after the scratch was made (zero) and in 24 h after that in fibroblasts (left graph) and keratinocytes (right graph) from the following experimental groups: nontreated (NT), exposed only to lipofectamine only (lipofectamine), transfected with scramble miR (scramble), transfected with synthetic miR-205 (miR-205 mimic), transfected with siRNA for miR-205 (siRNA), and, finally, transiently silenced for miR-205 and exposed to extracellular vesicles from mesenchymal stem/stromal cells (siRNA + EVs). Scale bar = 200 *μ*m. (b) Relative areas affected by the scratch wound in 24 h in all experimental groups. Data are presented as the mean ± standard deviation of three biological independent experiments. Statistical significance of differences is indicated as follows: ^∗∗^*P* < 0.01; ^∗∗∗^*P* < 0.001.

Finally, we investigate the migration effects of miR-205 overexpression and knockdown in fibroblasts and keratinocytes (Figure 8). The migration of fibroblasts was not affected by miR-205 overexpression or knockdown, but knockdown cells exposed to EVs showed faster migration. This result suggests that the migration of fibroblasts was independent of miR-205 and that EVs could have other molecules responsible for increasing fibroblast migration. On the other hand, migration of keratinocytes was increased by miR-205 overexpression. Accordingly, the knockdown of miR-205 made migration of keratinocytes similar to the levels of control groups, but the knockdown cells exposed to EVs had an increased migration. Taken together, these results suggest that miR-205 is important for keratinocyte migration, but, for the migration effect induced by EVs, its expression seems not to be essential.

Many studies are showing a complex role of miR-205 in many models [28]. For example, In HaCaT keratinocytes, miR-205 knockdown could promote migration in scratch wound healing assay. This could be due to the different cells used or because of the complex network of miR-205 [29]. In cancer, it acts either as an oncogene through facilitating tumor proliferation and initiation or as a tumor suppressor by inhibiting invasion and proliferation [28].

Considering the complexity of EV cargo, it is known that other molecules are present such as several growth factors and mRNAs. Perhaps many molecules could participate in the effects described in this paper. It is important, then, to fully elucidate the content of those EVs in terms of mRNAs, microRNAs, proteins, and other classes of molecules as to further explain the effects observed in this work.

## 4. Conclusion

In summary, our data suggest that cell migration and proliferation, key processes in skin wound healing, can be enhanced by exposure to MSC-derived EVs, which activate the AKT pathway in a miR-205-independent manner. Topical administration of a gel containing MSC EVs accelerated wound healing in an animal model, and this is an interesting finding as it opens perspectives for noninvasive model studies. For further development of this approach, it will be interesting to determine EV cargo and the molecules and mechanisms that mediate beneficial effects of MSC-derived EVs observed in this study.

## Supplementary Material

Supplementary figure 1 - Stem cell EVs induce tube formation in HUVEC cell line.

## Figures and Tables

**Table 1:** Extracellular vesicles isolated from MSCs conditioned by serum-free DMEM.

Characteristic	Sample data
Average size	135 nm
Concentration	1.89 × 10^8^ particles/mL
Spreading speed	1.201 nm·s^−1^
Sample viscosity	0.90 cP

Data were generated by the Nanoparticle Tracking Analysis software.
