# Supplementary material for: Extracellular Vesicles from Adipose-Derived Mesenchymal Stem/Stromal Cells Accelerate Migration and Activate AKT Pathway in Human Keratinocytes and Fibroblasts Independently of miR-205 Activity
Source: Stem Cells Int. 2017 Nov 5;2017:9841035. doi: 10.1155/2017/9841035 (PMC5735321; doi:10.1155/2017/9841035)

Supplementary data

Tube formation assay with HUVEC cell line

HUVEC cells were seeded in 24 wells plates previously coated with Matrigel® Matrix (Corning, cat. number: 354248) using 5 x 10^4 cells per well. Cells were then incubated at 37°C overnight in basic medium for HUVECs (50% DMEM + 50% DMEM F12 + 1% fetal bovine serum (Thermo Fisher Scientific). After 24 hours, VEGF (10ng/mL) or EVs (3,16 x 10^7 per well) were added according to each experimental group and incubated for 16 hours at 37°C. After this incubation period cells were stained with Calcein-AM (cat. number: C3100MP, Thermo Fisher Scientific) and photo documented in a QIClick camera (QImaging, Surrey, BC, Canada) controlled by Image-Pro Plus 7.0.1 (Media Cybernetcs, Rockville, MD, USA) coupled at IX70 Olympus microscope.

EVs promote tube formation in HUVEC cell line

Supplementary figure 1 shows images of an experiment designed to assess if EVs from MSCs could be able to stimulate the formation of tubes in HUVEC cells. Images suggest that EVs from human MSCs could induce tube formation in HUVEC cells compared to cells exposed only to culture medium. It is known that vascularization is an important step in wound healing [7]. These results suggest that the EVs could contribute for vascularization. More experiments are needed to investigate how EVs cargo can contribute for this process.

Supplementary figure 1 - Stem cell EVs induce tube formation in HUVEC cell line. Figure shows bright field images and fluorescent images (cells stained with Calcein-AM) of the following groups: negative control (cells exposed only to culture medium), cells exposed to VEGF or cells exposed to MSC EVs. The

results suggest that EVs could induce the formation of tubes in HUVEC cell lines. Experiments were carried out in three biological independent experiments. Scale Bar = 200 μm.

Supplementary Figure 1

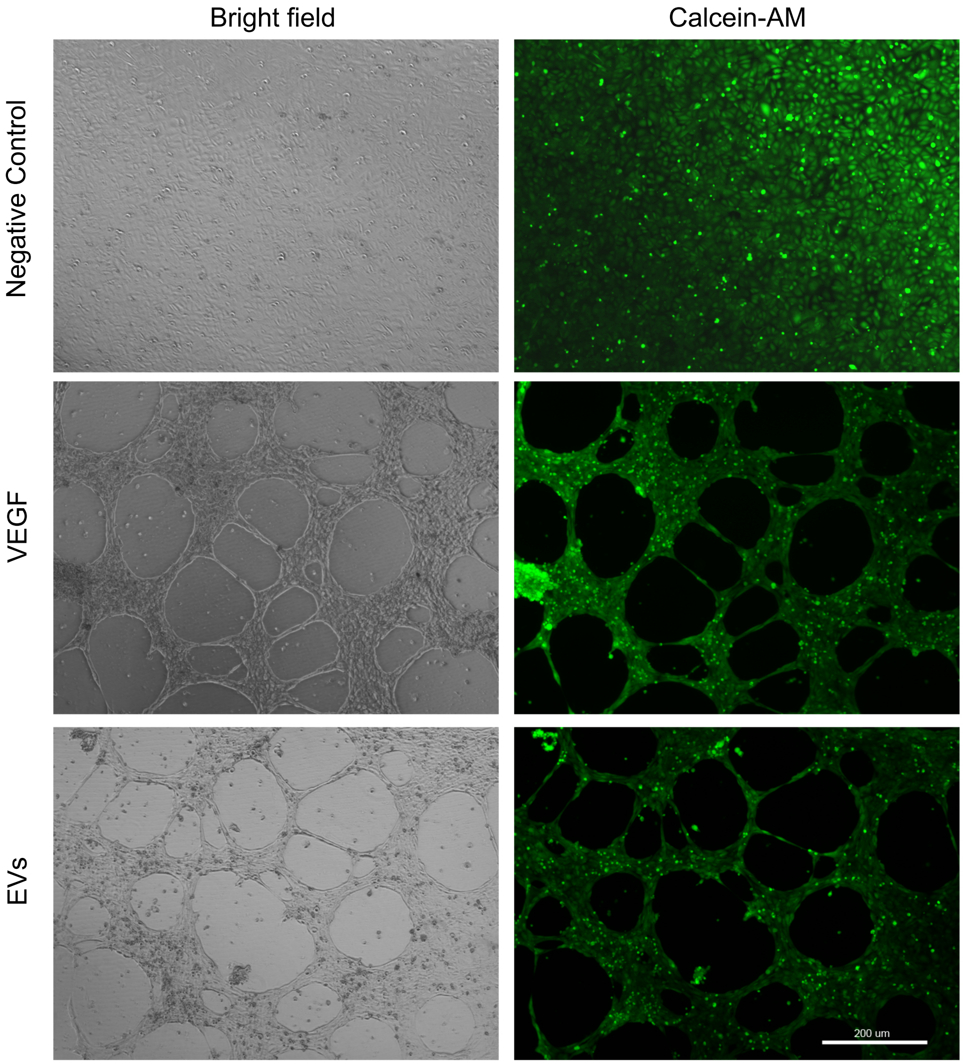

Supplement: Supplementary file 1 — Supplementary figure 1 - Stem cell EVs induce tube formation in HUVEC cell line. [file 9841035.f1.pdf]
